# Improved Cardiovascular Effects of a Novel Pomegranate Byproduct Extract Obtained through Hydrodynamic Cavitation

**DOI:** 10.3390/nu16040506

**Published:** 2024-02-10

**Authors:** Giada Benedetti, Lorenzo Flori, Jacopo Spezzini, Vincenzo Miragliotta, Giulia Lazzarini, Andrea Pirone, Cosimo Meneguzzo, Luca Tagliavento, Alma Martelli, Michele Antonelli, Davide Donelli, Cecilia Faraloni, Vincenzo Calderone, Francesco Meneguzzo, Lara Testai

**Affiliations:** 1 Department of Pharmacy, University of Pisa, 56126 Pisa, Italy; giada.benedetti@phd.unipi.it (G.B.); lorenzo.flori@farm.unipi.it (L.F.); jacopo.spezzini@phd.unipi.it (J.S.); alma.martelli@unipi.it (A.M.); vincenzo.calderone@unipi.it (V.C.); 2 Department of Veterinary Sciences, University of Pisa, 56126 Pisa, Italy; vincenzo.miragliotta@unipi.it (V.M.); giulia.lazzarini@phd.unipi.it (G.L.); andrea.pirone@unipi.it (A.P.); 3 Interdepartmental Research Centre of Ageing Biology and Pathology, University of Pisa, 56120 Pisa, Italy; 4 Centro per l’Integrazione della Strumentazione Scientifica dell’Università di Pisa (CISUP), Lungarno Pacinotti 43, 56126 Pisa, Italy; 5 HyRes Srl, Via Salvator Rosa 18, 82100 Benevento, Italy; cosimo.meneguzzo@hyres.it (C.M.); luca.tagliavento@hyres.it (L.T.); 6 Interdepartmental Research Center Nutrafood “Nutraceuticals and Food for Health”, University of Pisa, 56120 Pisa, Italy; 7 Department of Public Health, AUSL-IRCCS of Reggio Emilia, 42122 Reggio Emilia, Italy; michele.antonelli@ausl.re.it; 8 Department of Medicine and Surgery, University of Parma, 43121 Parma, Italy; davide.donelli@unipr.it; 9 Division of Cardiology, Azienda Ospedaliero-Universitaria di Parma, 43126 Parma, Italy; 10 Institute of Bioeconomy, National Research Council of Italy, Via Madonna del Piano 10, 50019 Florence, Italy; cecilia.faraloni@cnr.it (C.F.); francesco.meneguzzo@cnr.it (F.M.)

**Keywords:** ellagitannins, green extraction, hydrodynamic cavitation, hypertension, inflammation, cardiovascular risk, nutraceuticals

## Abstract

The healthy properties of pomegranate fruit, a highly consumed food, have been known for a long time. However, the pomegranate supply chain is still rather inefficient, with the non-edible fraction, whose weight is roughly half the total and is endowed with plenty of valuable bioactive compounds, either disposed of or underutilized. A novel extract obtained from non-edible byproducts (called PPE), using hydrodynamic cavitation, a green, efficient, and scalable technique, was investigated for its cardiovascular effects in vivo. PPE showed efficacy in an acute phenylephrine (PE)-induced hypertensive rat model, similar to the extract of whole fruit (PFE) obtained using the same extractive technique, along with good intestinal bioaccessibility after oral administration. Finally, when chronically administered for 6 weeks to spontaneously hypertensive rats, PPE was shown to significantly contain the increase in systolic blood pressure, comparable to the reference drug Captopril, and at a dose remarkably lower than the reported effective dose of ellagic acid. The extract from the non-edible fraction of the pomegranate fruit also showed good anti-inflammation and anti-fibrotic effects. The findings of this study, along with the extraction technique, could contribute to enhancing the value of the pomegranate supply chain, relieve the related environmental burden, and potentially improve public health.

## 1. Introduction

Pomegranate (*Punica granatum* L.) belongs to the family Punicaceae (also named Lythraceae) and originates from Asia, in the areas of Iran and Afghanistan. Nowadays, it is also cultivated in the Mediterranean region, Africa, North America, the Middle East, and Australia [1,2].

The plant is about 2–3 m tall and is characterized by thick branches and a tough wooden brown color bark. Pomegranate leaves appear green, glossy, and lanceolate, and flowers are characterized by 5–8 pink oval petals. The fruit (known by the name balausta) is represented by a large fleshy berry containing small red arils, composed of pulp (78%) and seeds (22%). Finally, the balausta is contained by a peel, which accounts for almost 50% of the whole fruit and is composed of a mesocarp which forms locules for the arils [3,4].

In ancient times, pomegranate was considered a sacred fruit and a symbol of fertility, luck, and abundance in different religions [5], while to date, the fruit has found application in medical use for its numerous beneficial effects. Pomegranate is indeed a source of organic acids, polyphenols—especially flavonoids, anthocyanins, tannins, and phenolic acids, including punicalagins, ellagic acid (EA), and gallic acid—tocopherols, vitamins, and terpenes [6]. Undoubtedly, the main phytochemicals involved in the beneficial effects are ellagitannins, among which punicalagins are the major constituents, which are metabolized into EA, and into urolithins by the gut microbiota at the gastro-intestinal level [3,7].

Pomegranate is consumed on a daily basis as a fresh fruit or as processed products—for example juice, jam, oil or infusion—but nowadays, supplementation with its components is also considered for a possible use as an adjuvant for the treatment of cardiovascular and non-cardiovascular diseases [2,3]. Indeed, pomegranate and its metabolites have interesting perspectives in the nutraceutical and nutritional fields and recent studies focused on their application in clinical use.

The main product obtained from the processing of pomegranate is juice; however, its non-edible parts can be used for infusion, tea, or spices due to their multiple properties [3,8,9,10,11]. Moreover, *P. granatum* seeds, rich in sterols and fatty acids, are used to obtain an oil rich in sterols and fatty acids [3,12].

However, the food industry produces a large amount of waste, consisting mainly of pomegranate peel and seeds, and research is scrambling to find new methods and applications for the use of such byproducts that are rich in bioactive compounds, such as new innovative packaging, cosmetic products, animal feed, or food additives, as well as to develop new extractive techniques to enable the sustainable exploitation of the byproducts [3,13,14].

In the literature, several extractive techniques of pomegranate byproducts were described, including supercritical fluid extraction, ultrasound-assisted extraction, microwave-assisted extraction, and aqueous ball milling [13,15,16,17,18]. Nevertheless, none of these technologies could meet the requirements of large-scale production. Thus, in recent years, researchers have been studying new methods to valorize waste products, exploiting efficient and green extraction techniques that are usable on an industrial scale.

In this context, hydrodynamic cavitation (HC) appears extremely promising, also due to the use of water as the only solvent [3,19,20,21]. This innovative technique, which allows for a substantial reduction in processing time and energy consumption while achieving high extraction yields, has already been verified in real-scale applications [22,23,24]. HC-based extracts of whole pomegranate fruits, including the peel and seeds, were recently tested in vitro for antiproliferative and apoptotic activity on breast cancer cells, with performances matching a commonly used drug [25]. It is noteworthy that 48 kg of fresh pomegranate fruit were extracted, with a biomass-to-water ratio of 1:2.1.

Another critical aspect of pomegranate extracts is the low oral bioavailability of ellagitannins, especially EA; indeed, it is reported to be poorly absorbed and endowed with a very low water solubility [26]. To overcome this challenging difficulty, which can jeopardize intestinal bioaccessibility, several formulations have been developed, such as cellulose ester solid dispersions [27], cyclodextrin nanosponges [28], or particle size reduction to microparticles or nanoparticles [29,30], as well as the creation of encapsulation biodegradable systems using polyvinyl alcohol or PVA associated with chitosan (80:20) [31,32,33].

This study investigates the performance of an HC-based extract obtained from whole pomegranate fruit (PFE), which was previously tested [25], and of a new HC-based extract obtained from pomegranate parts discarded after juice squeezing, including the peel and seeds (PPE), for their effects on hypertension-related cardiovascular risk. In particular, the acute anti-hypertensive effects of both extracts, as well as the pharmacokinetic profile and chronic anti-hypertensive effect of PPE are elucidated.

Our study aims to fill the most important gaps in the pomegranate processing industry: to recover and exploit the huge number of byproducts, and to obtain extracts that are useful for health and endowed with good intestinal bioaccessibility.

## 2. Materials and Methods

### 2.1. Manufacturing and Chemical Analysis of Pomegranate Extracts

PFE and PPE were obtained by means of the HC-based extraction of whole pomegranate fruits and pomegranate byproducts, respectively, in water only, using a semi-industrial-scale (200 L) HC pilot device optimized for food applications. The details of the HC-based extractor, comprising a closed hydraulic circuit with a centrifugal pump and a circular Venturi-shaped reactor as the key components, along with the meaning of the cavitation number as a measure of cavitation intensity and regime, were described in a previous study [21]. No active heat dissipation method was applied. Power and energy consumption were measured using a three-phase digital power meter (IME, Milan, Italy, model D4-Pd). The relative humidity of the used biological materials was assessed by drying an amount of 0.5 kg of biomass overnight at 75 °C using a commercial device (Electric Food Dryer, model LT 85, Kwasyo, Zhongshan, China).

#### 2.1.1. Extraction of Whole Pomegranate Fruit

Whole pomegranate fruits from the Wonderful One variety were purchased at a local market in the Apulia region, Salento area (southeastern Italy) in mid-November 2021, and processed the day after. The extraction process was described in a previous study [25], and here it is recalled in brief. A total of 48 kg of whole fresh pomegranate fruit, including peel and seeds, was ground in ice into coarse pieces with a maximum linear size of around 10 mm, then pitched in the HC device, where water was added until the volume including ice equaled 100 L. The relative humidity of the pomegranate fruits was 85%, thus, the dry biomass was 7.2 kg. The initial temperature was 21.5 °C, the processing time was 45 min, and the final temperature was 47 °C. The liquid PFE was filtered with a 200 µm sieve (stainless steel mesh) and stored in sterile bottles at −20 °C until analysis and use. The HC process was smooth, with a fairly constant cavitation number of 0.11 to 0.12, ensuring optimal cavitation yield [22].

#### 2.1.2. Extraction of Pomegranate Byproducts

The industrial pomegranate juice squeezing process was reconstructed as follows. Pomegranate fruits of the Wonderful One variety were purchased at a local market in the Apulia region, Salento area (southeastern Italy), in early October 2022, preserved in the dark at 4 °C and processed a few days later. Whole fruits were cut in half and squeezed manually using a commercial home tool (Pomegranate/citrus fruit squeezer Model AAA0000982081, Ilsa, Collegno, Italy), and the juice was discarded. All the other parts were retained for extraction, i.e., crown, peel composed of exocarp and mesocarp, endocarp and seeds. Such parts, which are the typical byproducts of pomegranate processing, were ground to coarse particles (5–20 mm) through a fruit mill (Model HP3, Polsinelli, Isola del Liri, Italy), and immediately processed.

A total of 47.3 kg of pomegranate byproducts was pitched in the HC device in less than 1 min, along with 108 L of water. The relative humidity of the pomegranate byproducts was 75%, thus, the dry biomass was 11.83 kg. The initial temperature of the extraction process was 28 °C, the processing time was 20 min, and the final temperature was 39 °C. The liquid PPE sample was filtered with a 50 µm polypropylene filter bag and stored in sterile bottles at −20 °C until analysis and use. The HC process was smooth, with a fairly constant cavitation number of 0.10 to 0.11, ensuring optimal cavitation yield [22].

The basic features of the extraction processes are shown in Table 1.

#### 2.1.3. Chemical Analysis of Pomegranate Extracts

The chemical analyses were performed on PFE and PPE after freeze drying. The identification and quantification of the various phenolic components was determined using HPLC-DAD as described by Romani et al. [34]. Analyses were carried out using a Varian ProStar 210 multisolvent pump and ProStar 335 photodiode array detector (Varian BV, Middelburg, The Netherlands). A Phenomenex Kinetex Phenyl-Hexyl 100 A 150 × 4.6 mm reverse-phase C18 column (Danaher Corporation, Torrance, CA, USA), with the same pre-column, was used for the separation and analysis at 25 °C. Water/acetic acid (99.9:0.1) and methanol/water CH_3_COOH made up the eluent (95:4.9:0.1). In particular, α and β punicalagin, and ellagic acid were identified by comparing the retention time and the spectrum with HPLC-grade standards (Sigma-Aldrich, St. Louis, MO, USA). The quantification was obtained using the calibration curve made with the relative standard. All analyses were performed in triplicate.

### 2.2. Animals Procedures

All the procedures involving male Wistar rats were carried out following the guidelines of the European Community Council Directive and in accordance with the Code of Ethics of the World Medical Association (Declaration of Helsinki, EU Directive 2010/63/EU for animal experiments). The experiments were authorized by the Italian Ministry of Health (authorization number 40/2023-PR, 10 January 2023). All the animals were housed in humidity and temperature-controlled rooms (22 °C and 50%, respectively) with 12 h light/dark cycles, water, and food availability ad libitum. All efforts to reduce and minimize the number of animals and their suffering were carried out. Animal studies were reported in compliance with the ARRIVE guidelines [35].

#### 2.2.1. In Vivo Acute Evaluation of the Anti-Hypertensive Effect

This study evaluated the effects of ellagic acid (EA), pomegranate fruit extract (PFE), pomegranate peel extract (PPE), and their respective vehicles (DMSO for EA and physiological solution for pomegranate extracts, 1 mL/kg) on blood pressure in 12-week-old male normotensive Wistar rats. Specifically, we carried out experiments on 5 animals for each group. Hypertension was acutely induced via a single intraperitoneal (i.p.) injection of phenylephrine (PE, 0.3 mg/kg). Briefly, under anesthesia with sodium thiopental (70 mg/kg, i.p.), the trachea was intubated for artificial ventilation (mod. 7025 Ugo Basile ventilator, Comerio, Italy; stroke volume 1 mL/100 g body weight; 70 strokes/min). An electrocardiogram (ECG, Mindray PM5000) continuously monitored vital functions. Moreover, a PE-20 tube was used to cannulate the carotid artery, which, connected to a pressure transducer (BIOPAC Apparatus, 2 Biological Instruments, Varese, Italy), was used to continuously record the systolic pressure (Psys) changes. Following a 30 min equilibration, PE was administered to induce hypertension. At the maximum hypertensive effect, EA (5, 25, 50 mg/kg), PFE (10, 50 and 100 mg/kg), PPE (10, 50 and 100 mg/kg), or their vehicles were orally administered, by gavage. Specifically, EA, PFE and PPE were dissolved in their vehicles and further dispersed in carboxymethylcellulose 4% before gavaging. When necessary, normal saline (0.1 mL), sodium citrate (3.8% *w*/*v*), or sodium thiopental (10 mg/kg) were injected intra-arterially. Psys was recorded for up to 90 min. Baseline Psys was measured before administering the pro-hypertensive agent. Changes in Psys, post-administration of EA, PFE, PPE, or vehicles, were expressed as a percentage of the PE-induced Psys at its plateau value (approximately 15 min after oral administration).

##### Data and Statistical Analysis

The experimental data were analyzed by a computer fitting procedure (GraphPad Prism 6.0, La Jolla, CA, USA) and expressed as mean ± SEM of five independent experiments. Blood pressure measurements were carried out in five animals per group. Student’s *t*-test was selected as statistical analyses. *p* < 0.05 was considered representative of significant statistical differences.

#### 2.2.2. Pharmacokinetic Profile

To investigate the pharmacokinetic profile of the PPE, and to speculate on the bioaccessibility and bioavailability of the active constituents, the time-course of the plasma levels of EA, punicalagin α or urolithin A were measured. Specifically, 12-week male Wistar rats (n = 5) were anaesthetized with sodium thiopental (70 mg/kg, i.p., Carlo Sessa, Milano, Italy). The trachea was intubated and connected to a rodent ventilator (mod 7025 Ugo Basile, Comerio, Italy) for artificial ventilation with room air (stroke volume, 1 mL/100 g body weight; 70 strokes/min). Electrocardiogram (ECG) was continuously measured by lead II (Mindray, PM5000; 2 Biological Instruments, Varese, Italy), to monitor the main vital functions. After a stabilization time of 15 min, PPE was orally administered. Blood samples (200 µL) were collected by carotid catheterization, at intervals of 5, 15, 30, 60, 120, 240, and 300 min after the administration of the extract. After the collection, each blood sample was immediately centrifuged (1100× *g*, 20 min, 25 °C) and the serum was separated (200 µL).

Each sample was deproteinized with acetonitrile:formic acid (HCOOH) (98:2, *v*/*v*) by vortexing for 2 min and ultrasonic bath for 10 min. The obtained mixture was submitted to centrifugation (15,000× *g* for 5 min) and the supernatant was led to dryness through a rotary evaporator Eppendorf Concentrator Plus (Eppendorf, Hamburg, Germany) at 30 °C for 2–3 h. Finally, dried samples were re-suspended in 100 µL MeOH and they were analyzed by LC/MS. The chromatographic system was a UHPLC-HRMS (ultra-high-performance liquid chromatography-high resolution mass spectrometry) composed of a chromatography UHPLC Vanquish Flex Binary coupled with a mass spectrometer detector (Orbitrap Q Exactive Plus) via a H-ESI source (Thermo Fisher Scientific Inc., Germering, Germany). For this technique, a Phenomenex EVO C18 column (100 × 2.1 mm, 2.6 µm) was used. The acquisition was carried out by setting a linear gradient using water 0.1% as mobile phase A and acetonitrile as phase B, both of them in the presence of formic acid 0.1%. The initial gradient started with 5% of solvent B in A, modifying the phase B quantity in 18% at 7 min, 28% at 17 min, 50% at 22 min, and 90% at 27 min for 1 min. At 29 min, the initial gradient was fixed again and kept under isocratic conditions for up to 33 min. The injection volume was 5 µL, and the flow rate was 0.5 mL/min at 35 °C. The parameters that have been set for the analysis are negative ionization, spray voltage of 3200 V, capillary temperature at 290 °C, S-lens RF level 50, sheath gas 28, and auxiliary gas 4.

#### 2.2.3. Evaluation of Anti-Hypertensive Effects in Chronic Hypertension Rat Model

Spontaneously hypertensive rats (SHRs), 7 weeks old and weighing 235 ± 4 g with a systolic blood pressure (Psys) of 191 ± 4 mmHg, were randomly divided into three groups (n = 5 for each group). The first group, the PPE group, received a supplement of 150 mg/kg PPE. The second group, the Captopril group, received Captopril at 20 mg/kg (Merck KGaA, Darmstadt, Germany), serving as a positive control due to its known anti-hypertensive properties. The third group, the vehicle group, received drinking water mixed with 0.2% (*v*/*v*) DMSO. Over the 6-week treatment period, the animals’ body weight, and their food and water intake were regularly monitored.

Twice a week Psys was measured at each animal using the sphygmomanometer approach, via a tail-cuff model, using a BP (BP-2000 Blood Pressure Analysis System, Series II, Visitech System, Apex, NC, USA) recorder. During this procedure, animals were collocated in cages on a heated plate (37 °C) for 10 min, then every 5 min, the blood pressure was measured three times.

At the end of the treatment period, after 9 h of fasting, rats were anaesthetized with sodium thiopental (100 mg/kg) via i.p. after measuring the glycemia parameter from the tail vein using the Glucocard G meter (Menarini Diagnostic, Florence, Italy). After testing their loss of consciousness, intracardiac blood was collected and stored in an EDTA tube (BD Vacutaine^®^, Dhaka, Bangladesh) to avoid coagulation. Complete lipid panel (triglycerides, total cholesterol, HDL, and LDL) and glycated hemoglobin levels were measured by Cobas b101 instruments (Roche Diagnostics^®^, Indianapolis, IN, USA). The remaining blood was centrifugated (1100× *g*, 15 min at room temperature) to obtain serum that was stored in Eppendorf at −80 °C. The heart was explanted and washed in PBS 1x at pH 7.4 (NaH_2_PO_4_ × H_2_O 18.6 mM; Na_2_HPO_4_ × H_2_O 74.6 mM; NaCl 1.5 M), then, it was dried and weighed to calculate heart weight/animal weight (g/kg) and left ventricle weight/animal weight (g/kg) as parameters of cardiac and ventricular hypertrophy.

Furthermore, the thoracic aorta was explanted and promptly cleared of surrounding tissues, including connective layers. It was then dissected into six rings, each 5 mm wide. The first ring was fixed in 4% paraformaldehyde for conducting the histological analysis, while the remaining rings were utilized for assessing endothelial integrity.

##### Endothelium Dysfunction

Aortic rings were suspended in baths containing Tyrode solution (composition: NaCl 136.8 mM; KCl 2.95 mM; CaCl_2_ × 2H_2_O 1.80 mM; MgSO_4_ × 7H_2_O 1.05 mM; NaH_2_PO_4_ × H_2_O 0.41 mM; NaHCO_3_ 11.9 mM; Glucose 5.5 mM), maintained at 37 °C and continually mixed with Clioxicarb (95% O_2_ and 5% CO) to simulate physiological conditions. Changes in tension were recorded using an isometric transducer (Grass FTO3; West Warwick, RI, USA), coupled with a preamplifier (Buxco Electronics; Wilmington, NC, USA) and the AcqKnowledge data acquisition software (version MP 100, BIOPAC Systems Inc.; Goleta, CA, USA).

After 40 min of stabilization, Norepinephrine (NE) (Merck KGaA, Darmstadt, Germany) 1 μM was added to the bath solution to induce a contraction. When the contraction reached a stable plateau, cumulative concentrations of Acetylcholine (Ach, Merck KGaA, Darmstadt, Germany) were added in a range of 1 nM–1 μM. Finally, after 30 min of stabilization in fresh Tyrode solution, aortic rings were contracted using KCl (Merck KGaA, Darmstadt, Germany) 60 mM to reach a stable value of maximum contraction for each ring.

##### Histological Analysis

The first aortic ring was fixed in formaldehyde 4% to preserve cellular morphology and avoid tissue degeneration; then, samples were paraffin-embedded and sectioned in 5 μm slices by microtome Leica RM 2055. After the rehydration of the sections, eosin/hematoxylin staining was performed to color nucleus and cytoplasm. Finally, the colored sections were observed using microscope Nikon Ni-e (Nikon Instruments Spa, Calenzano, Italy), scanned by Nano Zoomer Hamamatsu and analyzed by software Aperio Imagescope version 12.3.3 (Leica Biosystem, Buccinasco, Italy).

For each transversal section, 40 measurements of tunica media thickness have been recorded for each aortic ring.

##### Evaluation of TGF-β1 and IL-6 Content

Cardiac levels of IL-6 and serum levels of transforming growth factor beta 1 (TGFβ-1) were measured by ELISA assays, according to the manufacturing. Briefly, TGF-β1 serum levels were measured using the TGF-β1 (rat) ELISA kit (Boster Biological Technology, Pleasanton, CA, USA, catalog number: EK0514); IL-6 levels were assessed in heart tissue (left ventricle) using the IL-6 (rat) ELISA kit (4A Biotech, Beijing, PRC, catalog number CRE0005).

##### Data Analysis

Psys was reported as the mean of the two weekly measurements and analyzed using a computer fitting procedure (GraphPad Prism 8.0, La Jolla, CA, USA). Then, the Psys of the treated groups (with PPE and Captopril) was compared to the corresponding value of the vehicle group, analyzing their significant difference using Student’s *t*-test. The difference between the groups was considered statistically significant when *p* < 0.05 and indicated with an asterisk (*). For each group, the Psys mean was compared with the baseline value and statistical difference, calculated with paired Student’s *t*-test, and was indicated with the symbol §. The difference between the initial and final value was considered statistically different when *p* < 0.05. The vasorelaxant response induced by Acetylcholine was expressed as a percentage (%) of the contractile effect induced by NE. Potency index was expressed as pEC50, calculated as a negative logarithm of the molar concentration able to induce a 50% of the maximal vasorelaxant effect. In contrast, efficacy index, expressed as Emax, was referred to the maximum vasorelaxant effect obtained in that range of concentrations. The parameters for efficacy and potency were expressed as the mean ± SEM for each group.

The concentration-response curves of aortae from different treatment groups were analyzed using a two-way ANOVA followed by Bonferroni’s post-test. Differences between groups were considered significant at *p* < 0.05, marked with an asterisk (*). Hypertrophy parameters, aortic wall thickness, and levels of IL6 and TGFβ-1 were presented as the mean ± SEM for each group. All data were analyzed using GraphPad Prism 8.0, with Student’s *t*-test as the chosen statistical method. A *p*-value of less than 0.05 was used to denote statistical significance between groups.

## 3. Results

### 3.1. Ellagitannins Content of PFE and PPE

The overall content of α and β punicalagin and EA of freeze-dried PFE and PPE are shown in Table 2. It is worth noting that pomegranates contain many bioactive compounds other than ellagitannins, and their peels and juice show partially different compounds that significantly contribute to, for example, antioxidant activities [36].

### 3.2. Effect of PPE and PFE on Psys in PE-Induced Hypertensive Rats

**Figure 1:**
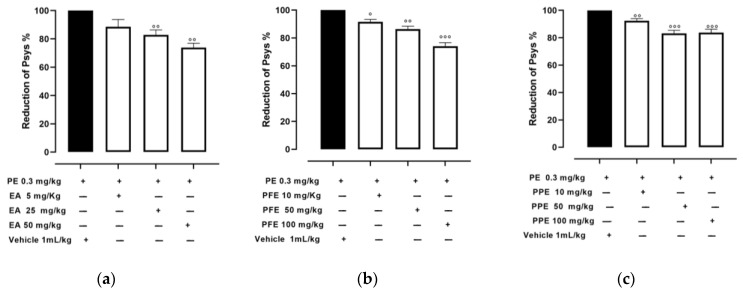
Effects of EA, PPE and PFE on blood pressure. Changes in Psys values, expressed in % vs. the maximal effect induced by injection of PE after oral administration of (**a**) vehicle (DMSO 1 mL/kg) or EA (5, 25, and 50 mg/kg), (**b**) vehicle (physiological solution 1 mL/kg) or PFE (10, 50, and 100 mg/kg), (**c**) vehicle (physiological solution 1 mL/kg) or PPE (10, 50, and 100 mg/kg) recorded until the plateau. The vertical bars indicate the SEM. Five different experiments were performed for each treatment. The symbol ° indicates a significant difference from Psys trend curve obtained after the administration of vehicle (° *p* < 0.05; °° *p* < 0.01, °°° *p* < 0.001).

Normotensive Wistar rats showed basal Psys of 133 ± 4 mmHg. The i.p. injection of PE raised it to 157 ± 4 mmHg; this hypertensive response corresponded to an increase of about 20% and remained almost stable for 90 min. The oral administration of the vehicle did not alter the basal Psys or the PE-induced hypertension state. In contrast, the oral administration of EA in amounts of 5, 25, and 50 mg/kg caused a significant dose-dependent decrease in Psys, showing a reduction of 11.3 ± 5%, 17.1 ± 4%, and 26.1 ± 3%, respectively, as shown in Figure 1a.

The oral administration of PFE (10, 50, and 100 mg/kg) showed hypotensive effects, reducing the PE-induced hypertensive peak of 8.5 ± 2%, 13.7 ± 2%, and 25.9 ± 3%, respectively, as shown in Figure 1b. Finally, hypotensive effect was observed also after the oral administration of PPE (10, 50, and 100 mg/kg), determining a decrease of the PE-induced hypertensive peak of 7.6 ± 2%, 16.7 ± 2%, and 16.3 ± 3%, respectively, as shown in Figure 1c.

### 3.3. Pharmacokinetic Profile

**Figure 2:**
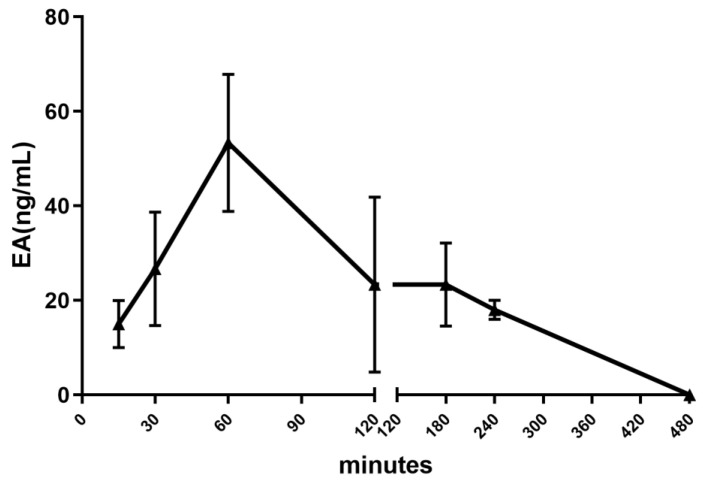
Pharmacokinetic profile of PPE after oral administration. Time-course of the plasma levels of EA, expressed as ng/mL, after oral administration of PPE. Blood samples were collected by carotid catheterization, at intervals of 5, 15, 30, 60, 120, 240, and 480 min after the administration of the extract.

The oral administration of PPE (100 mg/kg) led to a very early detection of EA in the systemic circulation (Figure 2); indeed, the plasma concentration of EA peaked 1 h after its administration, reaching the maximal concentration of 53 ± 14 ng/mL, as previously reported, but with higher doses of EA [26]. Four hours after the administration, the concentration of the tannin was negligible.

### 3.4. Anti-Hypertensive Effects of PPE Chronically Administered in SHR Rats

**Figure 3:**
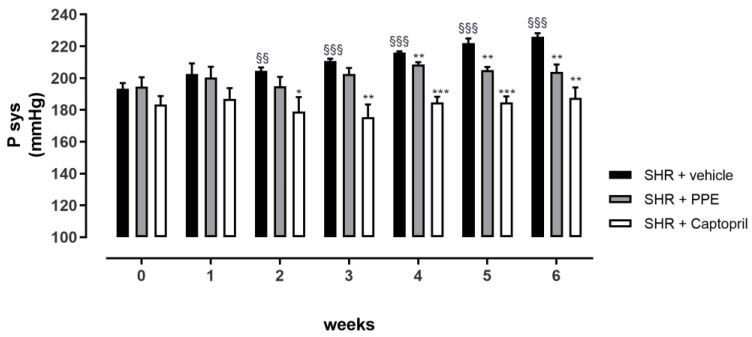
Antihypertensive effects of PPE in chronic protocol. Psys values measured during the period of treatment. The symbol * indicates a significant difference of treated group vs. vehicle group (* *p* < 0.05; ** *p* < 0.01; *** *p* < 0.001). The symbol § represents the significant difference between Psys measured week by week vs. the baseline value at week 0. (§§ *p* < 0.01; §§§ *p* < 0.001).

During the chronic treatment, animals did not unveil any evidence of toxicity, as shown in the trend of food and water intakes and ponderal parameters (data reported in Supplementary Information, Figure S1). As shown in Figure 3, SHRs of the vehicle group showed a progressive increase in the Psys value during the 6 weeks of treatment; in particular, from the 2nd week they showed a significant Psys increment compared to its own baseline value (193 ± 3 mmHg), and at the end of experimental protocol, the 6th week, Psys was 226 ± 2 mmHg, corresponding at an increment equal to 33 ± 4 mmHg. The Captopril group maintained a stable Psys value for all the treatment period, reaching a statistically significant difference from the vehicle group at the 2nd week; the last measurement of Psys (at the 6th week) revealed a value equal to 188 ± 7 mmHg, corresponding to an insignificant increment of 4±8 mmHg. Finally, the containment of Psys increments was also observed with PPE supplementation, in particular, the final Psys was 204 ± 5 mmHg (with an increment from the baseline equal to 9 ± 7 mmHg). It was not significantly different from its own baseline value (195 ± 6 mmHg) but was significantly different from the vehicle group’s value at the 6th week. Noteworthy, the final Psys levels resulting from the administration of PPE and Captopril were not significantly different.

#### 3.4.1. Glycemic and Lipidic Profile

Glycemia, glycated hemoglobin and total lipid panel were measured at the end of the chronic experimental protocol. As expected, no statistical differences were observed in these parameters from the three groups (data reported in the Supplementary Information, Table S1).

#### 3.4.2. Endothelium Dysfunction

The thoracic portion of aorta was used to evaluate endothelium integrity. In vehicle-SHR rats, the cumulative addition of acetylcholine produced—as expected—a concentration-dependent vasorelaxation, characterized by an Emax of 63 ± 2% and a pEC50 of 6.42 ± 0.06. Aortic rings from animals treated with Captopril showed an Acetylcholine vasorelaxation with Emax equal to 78 ± 3% and pEC50 of 6.90 ± 0.05; interestingly, this concentration-dependent curve differed significantly from the vehicle group aorta. These data suggested that, as reported in the literature, Captopril not only reduced Psys values, but also reduced the development of endothelial dysfunction, typical of the SHR model.

**Figure 4:**
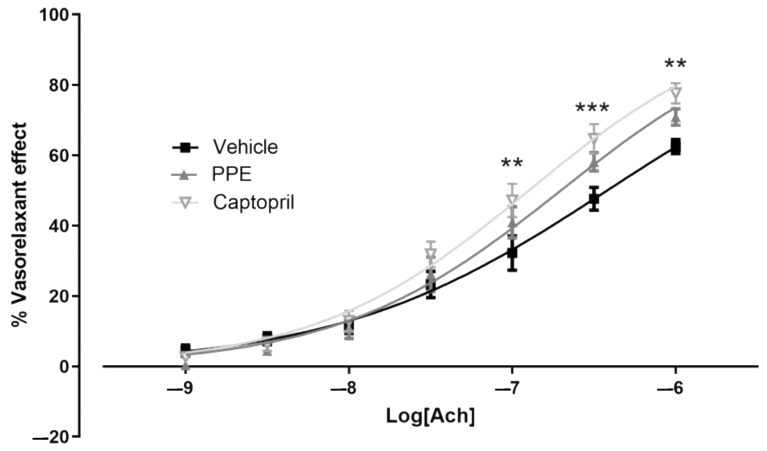
Preventive effects of PPE on endothelium layer. Concentration-dependent vasorelaxant response induced by Acetylcholine. It was expressed as a percentage (%) of the contractile effect induced by NE. The symbol * indicates significant differences between the Captopril group vs. the vehicle group (** *p* < 0.01; *** *p* < 0.001).

Finally, in the aortic rings from animals supplemented with PPE, acetylcholine produced a cumulative concentration-dependent vasorelaxation, which was lower than in the Captopril group. However, the Emax was 71 ± 2% and pEC50 was equal to 6.70 ± 0.047; even if the statistical analysis did not reveal a significant difference from the vehicle group, a trend of improvement in the vasorelaxant effect was observed (Figure 4).

#### 3.4.3. Histological Analyses

**Figure 5:**
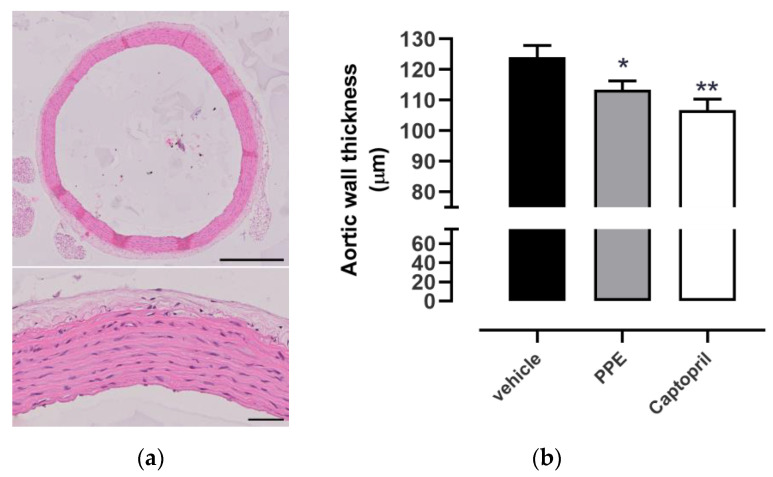
Effects of PPE on aortic wall thickness. (**a**) Representative pictures of a tunica media thickness used to carry out the histological measurement for each aortic ring. Scale bar is equal to 500 µm in the upper image and to 50 µm in the lower image. (**b**) Histograms showed the measurements of vascular thickness of aortic rings collected by SHR rats after each treatment. The symbol * indicates significant differences of treated group from vehicle group (* *p* < 0.05, ** *p* < 0.01).

Measurement of aortic wall thickness, by hematoxylin/eosin, as shown Figure 5a, highlighted that SHR rats had a mean of 124 ± 4 µm, indicative of a hypertrophy condition, if compared to normotensive rats. As expected, the Captopril group showed an aortic wall thickness equal to 107 ± 4 µm, lower than the vehicle one. PPE supplementation determined an intermediate effect between the vehicle and the Captopril group, with a value of 113 ± 3 µm. Statistical analysis revealed a significantly different value for Captopril as well as PPE compared to the vehicle group (Figure 5b).

#### 3.4.4. Cardiac and Ventricular Hypertrophy

The ponderal analysis of cardiac hypertrophy revealed a value of 3.85 ± 0.04 g/kg in the vehicle group and was indicative of hypertrophy if compared to the literature value [37,38]. A significant reduction in cardiac hypertrophy was observed in the Captopril group, where the mean value was 3.41 ± 0.06 g/kg, according to the ACE-inhibitor profile of this compound. As expected, this trend was also reflected in left ventricular hypertrophy (ventricular weight/animal weight), where the data obtained from Captopril rats (2.45 ± 0.04 g/kg) were significantly different from the vehicle value (2.92 ± 0.06 g/kg).

**Figure 6:**
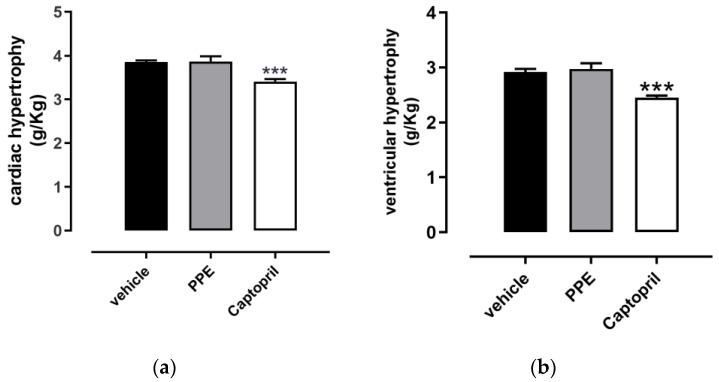
Effects of PPE on cardiac and ventricular hypertrophy. Histograms showed: (**a**) cardiac hypertrophy (expressed as a ratio between heart weight (g)/animal weight (kg)); (**b**) left ventricular hypertrophy (expressed as left ventricle weight (g)/animal weight (kg)). The symbol * indicates significant differences of the Captopril group vs. the vehicle group (*** *p* < 0.001).

Conversely, the PPE group did not reach the value of statistical significance either in cardiac hypertrophy (3.87 ± 0.12 g/kg) or left ventricular hypertrophy (2.98 ± 0.10 g/kg). The histograms of PPE supplementation were indeed completely comparable with the vehicle group (Figure 6).

#### 3.4.5. IL6 and TGF-β1 Content

**Figure 7:**
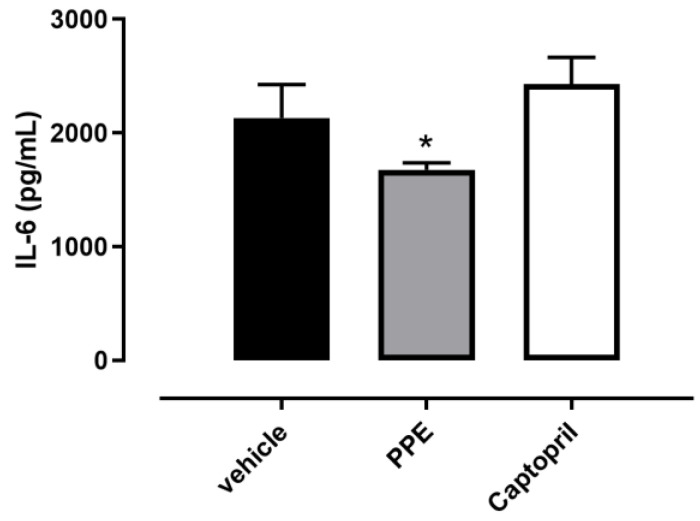
Effects of PPE on cardiac IL6 levels. Histograms showed the concentration of IL-6 in cardiac tissue collected by SHR rats after each treatment. The symbol * indicates significant differences between the PPE group and the vehicle group (* *p* < 0.05).

The IL-6 amount assessed in vehicle-treated rat hearts was 2130 ± 297 pg/mL, whereas the PPE value was 1676 ± 61 pg/mL, and it was significantly different compared to the vehicle group. Conversely, in the Captopril group, the level of this cytokine was not different from the vehicle group (2428 ± 236 pg/mL) (Figure 7).

**Figure 8:**
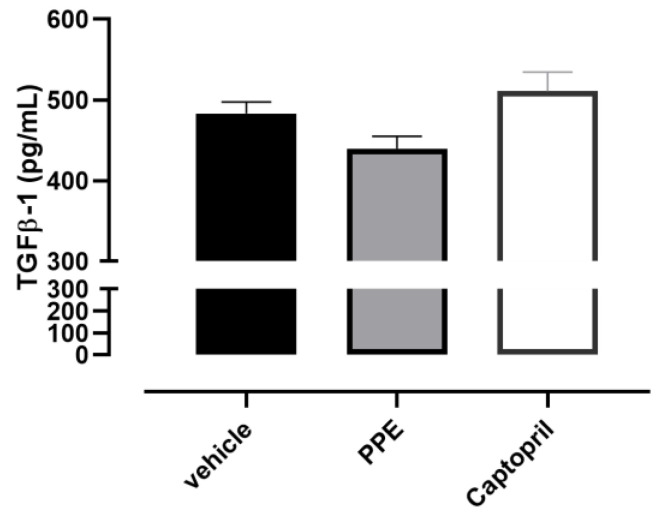
Effects of PPE on serum TGF-β1 levels. Histograms showed the concentration of TGF-β1 in serum samples collected by SHR rats after the treatment.

Finally, the serum TGF-β1 level in the vehicle group was 483 ± 15 pg/mL, indicative of pro-fibrotic stimuli probably induced by the chronic hypertension condition. Although no statistical significance was achieved, the PPE supplementation showed a trend of containment of this pro-fibrotic process, in fact, the levels of TGF-β1 were 438 ± 15 pg/mL. Instead, Captopril administration did not affect this parameter and a higher value was observed (551 ± 24 pg/mL) (Figure 8).

## 4. Discussion

Pomegranate is very interesting in the nutraceutical field for its beneficial effects in cardiovascular and non-cardiovascular diseases [3,39,40,41]. Pre-clinical and clinical evidence showed that the supplementation with pomegranate juice is correlated with a reduction in blood pressure, suggesting the prevention of cardiovascular risk associated with hypertension. Although the inhibition of the ACE enzyme seems to be the main action mechanism, further intracellular targets have been suggested, including the reduction of pro-inflammatory cytokines and the engagement of antioxidant mediators [42,43]. Such beneficial effects are estimated to be due to ellagitannins, available in pomegranate juice, but also abundant in the non-edible byproducts of the fruit, especially in the peel.

Of note, a large part of ellagitannins is represented by punicalagins, which gut microbiota transforms into EA at the gastric and colon level and into urolithins at the intestinal level [3,44,45]. A limitation to the clinical use of this interesting fruit is represented by the low bioavailability of its metabolites, among which is EA, which totals less than 1% [26]. Therefore, researchers are currently committed to improving bioavailability, creating new formulations or preparations, including through new or unconventional technologies that can combine high extraction yields and the preservation of nutrients and bioactive compounds, and have a reduced impact on the environment in terms of energy consumption and a lower or no reliance on synthetic solvents.

Another constraint hindering the sustainability of the pomegranate supply chain is the huge number of byproducts, mainly represented by the non-edible peel. In this work, two extracts of pomegranate were obtained using the HC technique, in particular, an extract was obtained from the whole fruit and a second extract from the byproducts (peel and seeds) was obtained after juice squeezing. Of note, both extracts contained a similar amount of ellagitannins and were endowed with optimal water solubility.

Both extracts showed anti-hypertensive effects when orally administered to PE-induced hypertensive rats. Of note, they were administered at the same dosage range and contained levels of ellagitannins that were approximately 20 times lower than in the experiments with EA; in fact, the lowest dose of EA (5 mg/kg) was achieved with a 100 mg/kg of extract; therefore, the other doses of extracts (10 and 50 mg/kg) contained even lower levels of ellagitannins. Interestingly, no significant differences arose between the two extracts (PPE and PFE), suggesting that the application of this extractive technique to the pomegranate byproducts may be considered suitable and useful for the full exploitation of the extracts.

To explore the extract obtained from pomegranate byproducts, which are the most interesting materials from a bioeconomy standpoint, we performed further experiments on the PPE.

From the pharmacokinetic analysis, it is possible to observe an absorption of EA, when the PPE was orally administered, while punicalagin α and urolithin A were not detectable in the blood. The absence of punicalagins in the blood is in accordance with the literature [46], and it is likely due to the extensive pre-systemic metabolization of these compounds; whereas urolithins need a longer time to appear in the blood. Likewise, during a chronic treatment, this metabolite may contribute to the beneficial effects of pomegranate.

Moreover, PPE, chronically supplemented to SHR rats, was able to contain the Psys increment for the entire treatment period (6 weeks), contrasting the pathophysiological increment observed in the vehicle group. Such a profile was slightly lower than in the Captopril group, used as a positive reference drug. This result is important in a nutraceutical perspective, aimed at the maintenance of homeostasis and the prevention of hypertension condition. As expected, the metabolic parameters represented by fasting blood glucose levels and lipidic panel were not altered in SHRs belonging to the vehicle group and the administration of PPE or Captopril did not significantly change the normal metabolic profile of this pre-clinical animal model.

It is well-known that chronic hypertension induces endothelial damage over time, affecting its structural and functional role. This endothelial dysfunction is accompanied by vascular dysfunction, characterized by the thickening and stiffening of vascular walls, and determining a risk for organs, such as the heart, the kidneys, and the brain [47,48,49,50]. From the evaluation of endothelium-mediated vasorelaxation evoked by acetylcholine in aortic rings from the PPE-treated animals, the partial protection of endothelial dysfunction, even if less than in Captopril-treated animals, emerged. These results reflected the histological analyses obtained in aortic rings embedded in paraffin and stained with hematoxylin/eosin.

With regard to the impact of nutraceutical supplementation on the heart and on myocardial remodeling, no anti-hypertrophic effect was observed in SHRs receiving PPE for 6 weeks. Conversely, the Captopril group showed a significant reduction in cardiac hypertrophy if compared to the vehicle group, which suggests, according to the literature, an inversion of the remodeling processes. Interestingly, PPE, but not Captopril, reduced the cardiac levels of pro-inflammatory cytokines IL6 and the serum amount of pro-fibrotic TGF-β1.

A limitation of this study lies in the characterization of the pomegranate extracts, which was limited to ellagitannins and did not allow, for example, to safely explain the apparently more intense anti-hypertensive effect of PFE at the highest considered dose of 100 mg/kg. Even if the scope of this study focused on the comparison with EA in acute conditions and the reference drug Captopril in chronic conditions, future research should address a more complete characterization and explore the distinct contribution of other compounds, such as flavonoids and anthocyanins.

Further research is warranted in regard to the structural features of the PPE, which could underlie some of its distinctive properties, such as the bioaccessibility and the improved cardiovascular profile. Based on the abundance of pectin in the pomegranate peel [51], such properties could have been boosted by an IntegroPectin-like structure of the extract [52], which is also deemed to have contributed to the cardioprotective effects of a HC-based extract of grapefruit by-products [53].

## 5. Conclusions

Taken together, these results suggest that PPE is an innovative extract, highly water-soluble and characterized by good enteric bio-accessibility, effective in counteracting hypertension and preventing endothelial and vascular dysfunctions associated with hypertension, as well as showing significant anti-inflammatory and anti-fibrotic effects. Furthermore, the origin of the aforementioned extract from byproducts of the pomegranate supply chain, and the sustainability of the extraction technique, allow for creating an interesting opportunity to bring further value to the supply chain, while alleviating the environmental burden of the disposal of the byproducts, potentially contributing to public health.

## Figures and Tables

**Table 1:** Process time, temperature range, and specific energy (kWh per kg of dry raw material), for the manufacturing of PFE and PPE.

Extract	Time (min)	Temperatures (°C)	Specific Energy (kWh/kg)
PFE	45	21.5–47.0	0.720
PPE	20	28.0–39.0	0.216

**Table 2:** Overall α and β punicalagin and ellagic acid contents of PFE and PPE.

Extract	α + β Punicalagin (mg/g)	Ellagic Acid (mg/g)
PFE	38.96 ± 2.71	1.03 ± 0.05
PPE	48.48 ± 5.45	3.07 ± 0.19

## Data Availability

Dataset available on request from the authors.

## Supplementary Materials

The following supporting information can be downloaded at: https://www.mdpi.com/article/10.3390/nu16040506/s1, Figure S1. Ponderal parameters; Table S1. Blood parameters of Total Cholesterol, Triglycerides and Glycemia.
