# Supplementary material for: Improved Cardiovascular Effects of a Novel Pomegranate Byproduct Extract Obtained through Hydrodynamic Cavitation
Source: Nutrients. 2024 Feb 10;16(4):506. doi: 10.3390/nu16040506 (PMC10893178; doi:10.3390/nu16040506)
Supplement: Supplementary file 1 [file nutrients-16-00506-s001.zip › nutrients-2845863-supplementary.pdf]

Improved Cardiovascular Effects of a Novel Pomegranate Byproduct Extract Obtained through Hydrodynamic Cavitation

Giada Benedetti [1], Lorenzo Flori [1], Jacopo Spezzini [1], Vincenzo Miragliotta [2,3,4], Giulia Lazzarini [2], Andrea Pirone [2], Cosimo Meneguzzo [5], Luca Tagliavento [5], Alma Martelli [1,3,6], Michele Antonelli [7], Davide Donelli [8,9], Cecilia Faraloni [10], Vincenzo Calderone [1,3,6], Francesco Meneguzzo [10,†] and Lara Testai [1,3,4,6,*,†]

1 Department of Pharmacy, University of Pisa, 56126 Pisa, Italy; giada.benedetti@phd.unipi.it (G.B.); lorenzo.flori@farm.unipi.it (L.F.); jacopo.spezzini@phd.unipi.it (J.S.); alma.martelli@unipi.it (A.M.); vincenzo.calderone@unipi.it (V.C.)
2 Department of Veterinary Sciences, University of Pisa, 56126 Pisa, Italy; vincenzo.miragliotta@unipi.it (V.M.); giulia.lazzarini@phd.unipi.it (G.L.); andrea.pirone@unipi.it (A.P.)
3 Interdepartmental Research Centre of Ageing Biology and Pathology, University of Pisa, 56120 Pisa, Italy
4 Centro per l'Integrazione della Strumentazione scientifica dell'Università di Pisa (CISUP), Lungarno Pacinotti 43, 56126 Pisa, Italy
5 HyRes Srl, via Salvator Rosa 18, 82100 Benevento, Italy; cosimo.meneguzzo@hyres.it (C.M.); luca.tagliavento@hyres.it (L.T.)
6 Interdepartmental Research Center Nutrafood "Nutraceuticals and Food for Health", University of Pisa, 56120 Pisa, Italy
7 Department of Public Health, AUSL-IRCCS of Reggio Emilia, 42122 Reggio Emilia, Italy; michele.antonelli@ausl.re.it
8 Department of Medicine and Surgery, University of Parma, 43121 Parma, Italy; davide.donelli@unipr.it
9 Division of Cardiology, Azienda Ospedaliero-Universitaria di Parma, 43126 Parma, Italy
10 Institute of Bioeconomy, National Research Council of Italy, Via Madonna del Piano 10, 50019 Florence, Italy; cecilia.faraloni@cnr.it (C.F.); francesco.meneguzzo@cnr.it (F.M.)
* Correspondence: lara.testai@unipi.it
† These authors contributed equally to this work.

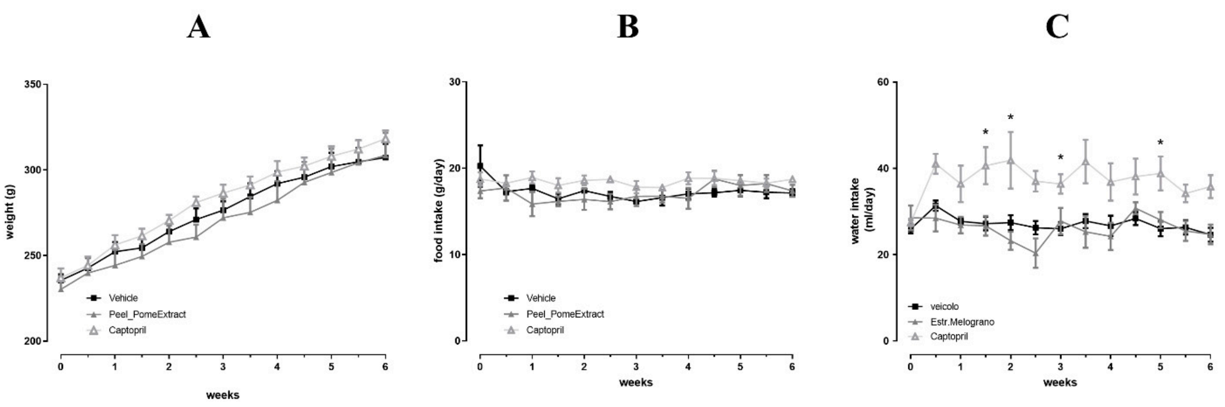

Figure S1. In this figure graphics show the trend of ponderal parameters (A), food (B) and water intakes (C)

	**Vehicle**	**PomeExtract**	**Captopril**
Total Cholesterol (mg/dl)	58.8 ± 3.2	60.0 ± 2.9	57.3 ± 2.7
Triglycerides (mg/dl)	49.5 ± 2.7	51.0 ± 2.5	47.5 ± 2.2
Glycemia (mg/dl)	63.8 ± 4.5	61.3 ± 3.8	63.0 ± 4.8

Table S1. In this table blood parameters of Total Cholesterol, Triglycerides and Glycemia are shown. All values are expressed as mg/dl. No relevant difference is observed in samples of animals from the three different treatments.
